# Benefit of Laughter

**Published:** 1872-01

**Authors:** 


### BENEFIT OF LAUGHTER.

It is said by good medical authority that there is not the remotest corner or little inlet of the minute blood vessels of the human body that does not feel some wavelet from the convulsion occasioned by good hearty laughter, and also that the "central man" or life principle is shaken to its innermost depths, sending new tides of life and strength to the surface, and thus materially tending to insure good health to the persons who indulged therein. The blood moves more rapidly—probably caused by some chemical or electric modification occasioned by the convulsion—and conveys a different impression to all the organs of the body as it visits them on that particular mystic journey, when the man is laughing, from what it does at other times.

For this reason every good hearty laugh, in which persons indulge tends to lengthen his life, conveying, as it does, new stimulous to the vital forces. We doubt not the time will come when physicians, conceding more importance than they do to the influence of the mind upon the vital forces of the body, will prescribe to the torpid and melancholy patient a certain number of hearty peals of laughter, to be undergone at stated periods, and believe that they will, in so doing, find the best and most effecttive method of producing the required effect upon the patient. Our advice to all is, indulge in good, hearty, soulful laughter, when the opportunity offers, and if you do not derive material benefit therefrom, charge us with uttering false principles of *mataria medica.*

A JOKE.—A prominent physician in Madison Avenue, New York, was somewhat annoyed recently, to observe tacked to his office door, the other morning, a tin sign (evidently transferred by some jester from a barber shop), which read: "Gentlemen Wishing Dying Done Should Apply within."

A WIFE TEXT—"The right man in the right place"—a good husband at home in the evening.

